# Supplementary material for: Photocatalytic Degradation of Diclofenac Using Al2O3-Nd2O3 Binary Oxides Prepared by the Sol-Gel Method
Source: Materials (Basel). 2020 Mar 16;13(6):1345. doi: 10.3390/ma13061345 (PMC7142804; doi:10.3390/ma13061345)
Supplement: Supplementary file 1 [file materials-13-01345-s001.pdf]

materials

Supplementary Information

Article

Photocatalytic Degradation of Diclofenac Using Al_2O_3-Nd_2O_3 Binary Oxides Prepared by the Sol-Gel Method

José Eduardo Casillas [1], Jorge Campa-Molina [2], Francisco Tzompantzi [3], Gregorio Guadalupe Carbajal Arízaga [4], Alejandro López-Gaona [3], Sandra Ulloa-Godínez [2], Mario Eduardo Cano [1] and Arturo Barrera [1,*]

1 Departamento de Ciencias Básicas, Centro Universitario de la Ciénega, Universidad de Guadalajara, Av. Universidad, No. 1115, C.P. 47820, Ocotlán, Jalisco, México
2 Departamento de Electrónica, Universidad de Guadalajara, Marcelino García Barragán 1422, C.P. 44430, Guadalajara, Jalisco, México
3 Departamento de Química, Universidad Autónoma Metropolitana – Iztapalapa, San Rafael Atlixco 189, C.P. 09340, Ciudad de México, México
4 Departamento de Química, Universidad de Guadalajara, Marcelino García Barragán 1422, C.P. 44430, Guadalajara, Jalisco, México
* Correspondence: arturo.barrera@cuci.udg.mx or arturobr2003@yahoo.com.mx

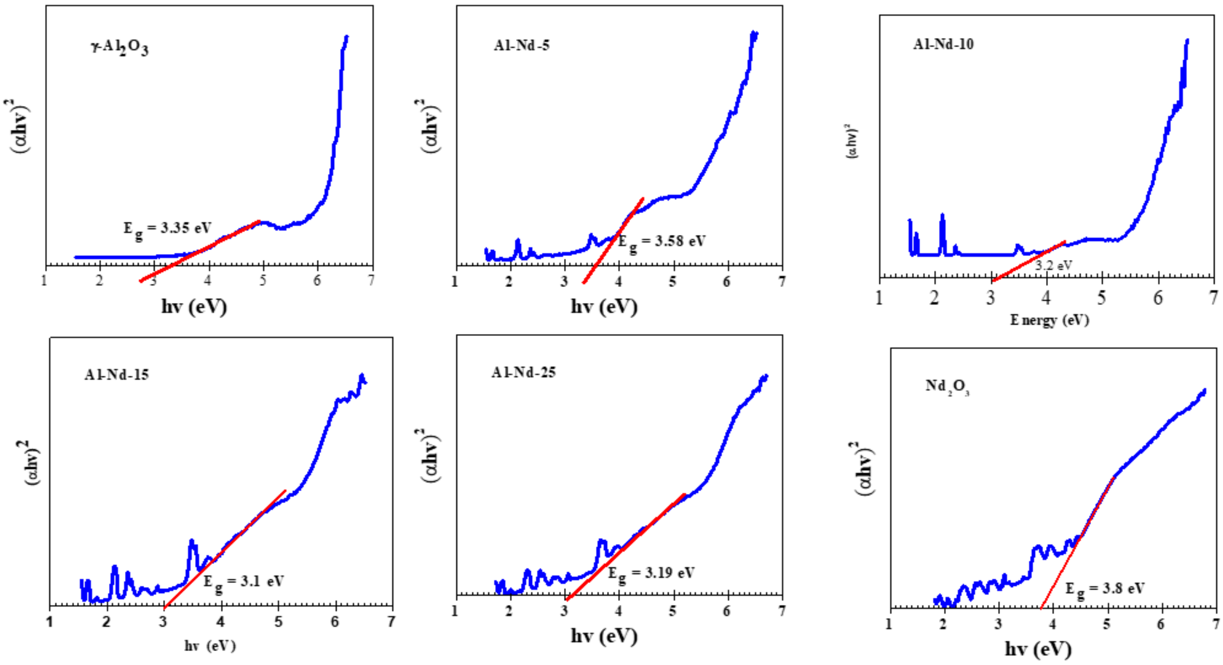

Figure S1. $(\alpha h\nu)^2$ vs. Energy plot for the calculation of the band gap energy of Al-Nd-*x* binary oxides prepared by the sol-gel method.
